# The Role of the Rat Medial Prefrontal Cortex in Adapting to Changes in Instrumental Contingency

**DOI:** 10.1371/journal.pone.0033302

**Published:** 2012-04-04

**Authors:** Etienne Coutureau, Frederic Esclassan, Georges Di Scala, Alain R. Marchand

**Affiliations:** 1 Institut de Neurosciences Cognitives et Intégratives d'Aquitaine (INCIA), Université de Bordeaux, Talence, France; 2 Institut de Neurosciences Cognitives et Intégratives d'Aquitaine (INCIA), CNRS, UMR 5287, Talence, France; University of Chicago, United States of America

## Abstract

In order to select actions appropriate to current needs, a subject must identify relationships between actions and events. Control over the environment is determined by the degree to which action consequences can be predicted, as described by action-outcome contingencies – i.e. performing an action should affect the probability of the outcome. We evaluated in a first experiment adaptation to contingency changes in rats with neurotoxic lesions of the medial prefrontal cortex. Results indicate that this brain region is not critical to adjust instrumental responding to a negative contingency where the rats must refrain from pressing a lever, as this action prevents reward delivery. By contrast, this brain region is required to reduce responding in a non-contingent situation where the same number of rewards is freely delivered and actions do not affect the outcome any more. In a second experiment, we determined that this effect does not result from a different perception of temporal relationships between actions and outcomes since lesioned rats adapted normally to gradually increasing delays in reward delivery. These data indicate that the medial prefrontal cortex is not directly involved in evaluating the correlation between action-and reward-rates or in the perception of reward delays. The deficit in lesioned rats appears to consist of an abnormal response to the balance between contingent and non-contingent rewards. By highlighting the role of prefrontal regions in adapting to the causal status of actions, these data contribute to our understanding of the neural basis of choice tasks.

## Introduction

Decision making requires adequate integration of actions with respect to their goal. A number of studies have demonstrated that this process depends on the identification of causal relationships between actions and events [1], which amounts to contingency learning. Contingency is usually defined as the difference between the probability to observe a given outcome in the presence of a given action and the same probability in the absence of this action.

An increasing body of evidence points to a role of prefrontal regions in the representation of contingencies. In particular, activity within prefrontal areas in both primates and rodents is related to the acquisition and updating of contingency [2], [3], [4]. In rodents, the medial prefrontal cortex (mPFC) contributes to the learning of instrumental contingencies in animals pressing a lever for a food reward [5], [6]. This research has established that rats with damage to the mPFC learn the task at a normal rate [7], [8], but that their response is insensitive to manipulations of consequences such as contingency degradation i.e. weakening the correlation between food delivery and lever pressing [5], [6], [9], [10]. Dopaminergic mechanisms also appear to be involved since lesions of dopaminergic terminals in the mPFC alter normal adaptation to contingency degradation [11].

The mechanisms responsible for these effects however remain poorly known. In a standard contingency degradation procedure, the outcome is equally probable in the presence or absence of action (see [12]). Thus both the causal and temporal relationship between action and outcome are altered. Normal rats, but not mPFC-lesioned rats, respond to this new situation by reducing their lever-pressing rate. This deficit might result from either the degree of control over the outcome or the temporal relationship between response and outcome. The present study therefore aims at elucidating this issue.

First, the mPFC might be required for adaptation when there is no clear relationship between the action and the outcome, i.e. under conditions of low or null contingency. We tested this hypothesis by comparing the performance of previously trained mPFC-lesioned animals in two contingency conditions. In a first condition, classically called omission (e.g. [13]), the animals had to refrain from pressing the lever for a fixed time (20 s) in order to obtain the food reward. Thus, although food could not be obtained by lever pressing, a relationship between action and outcome was preserved (negative contingency). In a second condition, reward delivery was independent of lever pressing, a non-contingent situation.

Second, since a condition of degraded contingency is characterized by a variable time interval between lever-press and response delivery, the deficit of mPFC-lesioned rats might result from an altered perception of the temporal relationship between action and reward delivery. We evaluated this hypothesis in a second experiment under delayed reward conditions that gradually disrupted the contiguity between lever press and reward.

## Experiment 1

### Materials and Methods

#### Ethics statement

All procedures involving animals and their care conformed the institutional guidelines that comply with international (Directive 86-609, November 24, 1986, European Community) and national (council directive 87-848, october 19, 1987, *Ministère de l'Agriculture et de la Forêt, Service Vétérinaire de la Santé et de la Protection Animales*) laws and policies. They adhered to protocols approved by Région Aquitaine Veterinary Services (Direction Départementale de la Protection des Animaux, approval ID: A37-063). E.C. holds permission for animal experiments no. 33 06 008 from *Ministère de l'Agriculture et de la Forêt*. Surgery was performed under ketamine+xylazine anaesthesia (Expt. 1) or isoflurane anaesthesia (Expt. 2). Following surgery, animals were daily weighted and observed to detect and minimize pain or discomfort.

#### Subjects

Thirty two male, Long Evans rats obtained from Centre d'Elevage Janvier (France) were used. Rats were housed in pairs and accustomed to the laboratory vivarium for one week. The vivarium was maintained at 21°C±1°C with the light on from 7 a.m. to 7 p.m. All experiments were carried out during the light portion of the cycle. Following recovery from surgery animals were maintained at about 90% of free feeding weight (340–405 g) by providing the animals once daily with 15 g rodent formula (laboratory chow, Purina).

#### Surgery

The rats were anaesthetised using a mixture of ketamine (90 mg/kg) and xylazine (10 mg/kg) and then placed in a Kopf stereotaxic frame (Kopf instruments, Tujunga, CA) in a flat skull position. Neurotoxic lesions were performed using multiple NMDA micro-injections. The bone above the injection sites was removed using a high-speed drill. NMDA (Sigma-Aldrich) 40 mM in PBS (pH = 7.4) was injected into the brain through a glass pipette glued onto the end of the needle of a 5-µl Hamilton syringe held with a microinjector (Imetronic, Pessac, France). For the lesioned group (mPFC, n = 16), 0.1 µl of NMDA was infused in the medial prefrontal cortex at the following coordinates (in mm from Bregma): A-P (antero-posterior)+3.8, L (lateral)±0.6, V (ventral) −3.8; A-P +3.2, L ±0.6, V −3.6; A-P +3.0, L ±0.6, V −5.4; A-P +2.5, L ±0.6, V −3.4. Injections were made at a rate of 0.10 µl/min then the pipette was left in place for 5 min to allow diffusion of the solution into the tissue. The control group (SHAM, n = 16) was given a similar surgical procedure but the dura was simply breached using a standard needle and no injection was given. All subjects recovered for a period of at least 7 days after surgery with *ad lib* access to food and water. Animals were then individually handled for 5 min on each of 3 days, after which the food deprivation schedule and behavioural experiments were initiated.

#### Apparatus

Eight identical (40 cm wide×30 cm deep×35 cm high) operant chambers (Imetronic, Pessac, France) were used in this experiment. They were individually enclosed in ventilated, sound- and light-attenuating wooden cubicles. Each chamber had a stainless-steel grid floor above a sawdust tray. The left panel of the chamber featured a recessed food magazine in its centre and a retractable lever (2×4×1 cm) located on the left of the magazine, 7 cm above the grid floor. An external food dispenser delivered calibrated rodent formula pellets (Bioserv, NJ) into the magazine. All experiments were designed and controlled from a PC with real-time software (Imétronic, Pessac, France).

#### Behavioural procedures

During two daily magazine training sessions, rats were accustomed to the operant chambers and allowed to consume the food pellets used as rewards. During each 30 min session, 30 food pellets were delivered into the magazine at pseudo-random intervals. No lever was presented at this stage.

**Figure 1:**
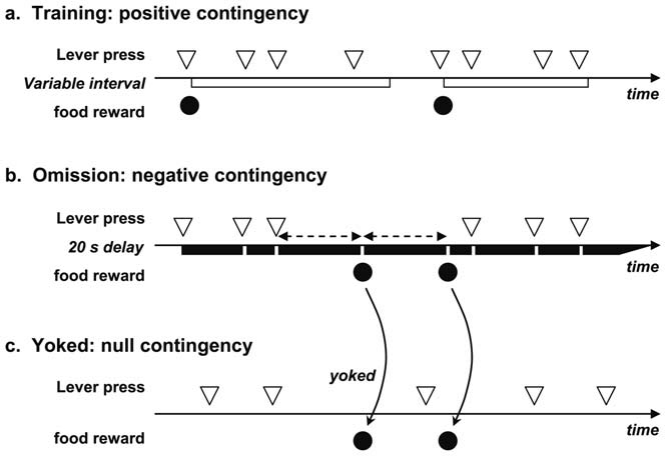
Time line representation of the contingency conditions. A) During instrumental training (positive contingency), the lever becomes inactive for a variable interval (white rectangle) following each reward delivery. The first lever press after this interval triggers an immediate reward. No reward occurs in the absence of lever press (positive contingency). B) During omission training, rewards are delivered following a 20 s delay without lever press (black rectangle). A lever press during the delay resets the delay. Consecutive rewards are delivered at 20 s intervals in the absence of lever press activity (negative contingency). C) During yoked training, rewards are synchronized to the rewards of another rat trained in omission, regardless of the yoked rat's activity. Rewards may occur at any time with respect to lever presses (null contingency).

For initial lever press training, each training session began with the illumination of the houselight and insertion of the lever and ended with the retraction of the lever and turning off of the houselight. The rats were first trained for 2 sessions under a fixed interval 20 s (FI-20s) schedule, in which food pellets could be obtained every 20 s by pressing the lever. A session ended as soon as 50 rewards were earned or after 45 minutes had expired. The rats were then switched to a single session of variable-interval 30 s schedule (VI-30 range 7.5–75 s), under which a pellet became available every 30 s on average if the rat then pressed the lever, then to 4 sessions under a variable-interval 60 s schedule (VI-60 range 15–150 s). These sessions ended as soon as 30 rewards were earned or 45 minutes had expired. Throughout instrumental training, although only some of the lever presses were rewarded, food was never delivered in the absence of lever pressing, thereby ensuring that a positive contingency was in effect (Figure 1a).

After the initial training, the action-outcome contingency was changed. Each group of rats was divided in two and each half was switched to one of two contingency conditions, either negative or null (Figure 1b, c). Within each lesion group, rats were associated in pairs, corresponding to the two conditions. Within each pair, the rat in the negative contingency condition (omission schedule) obtained a pellet whenever 20 s had elapsed without the rat pressing the lever. The other rat in the pair (yoked) received pellets delivered at exactly the same instants, irrespective of its behaviour (null contingency condition). Thus, in the negative contingency condition, food deliveries occurred well apart from lever pressing, i.e. 20 s after the previous lever press or pellet delivery, whereas in the null contingency condition, reward delivery could occur at any time with respect to lever pressing. Importantly, this procedure equated the amount of food pellets delivered in each group.

On the following day, the rats were returned to the operant chambers for a 30 min test session, in which the lever was inserted, but no food was delivered.

#### Histology

After behavioural testing, animals received a lethal dose of sodium pentobarbital and were perfused transcardially with saline (0.9%) followed by 10% buffered formalin. The brains were removed and post-fixed in a formalin-saccharose 30% solution for 2 days, then were frozen and cut into 40 µm-thick coronal sections with a freezing microtome (−20°C). The sections were collected onto gelatin-coated slides and dried before being stained with thionine. Histological analysis was performed under the microscope by an experimenter (F.E.) blind to lesion condition. Sections were examined for gross morphological changes, gliosis and scarring. The extent of lesions was reconstructed in reference to the atlas of Paxinos and Watson [14].

#### Data analysis

Rates of lever pressing and magazine entries were calculated over blocks of 5 min of training and over the whole session of test. Statistical analyses were performed on StatView ® software (SAS Institute Inc.) with ANOVA and Student-Newman-Keuls *post-hoc* tests, using lesion (Sham, mPFC) and condition (negative *vs.* null contingency) as between subject factors and blocks as repeated measures when appropriate. The alpha value for rejection of the null hypothesis was 0.05 throughout. Complementary analyses and modelling of action sequences from this experiment are provided in a related paper [7].

### Results

#### Histology

**Figure 2:**
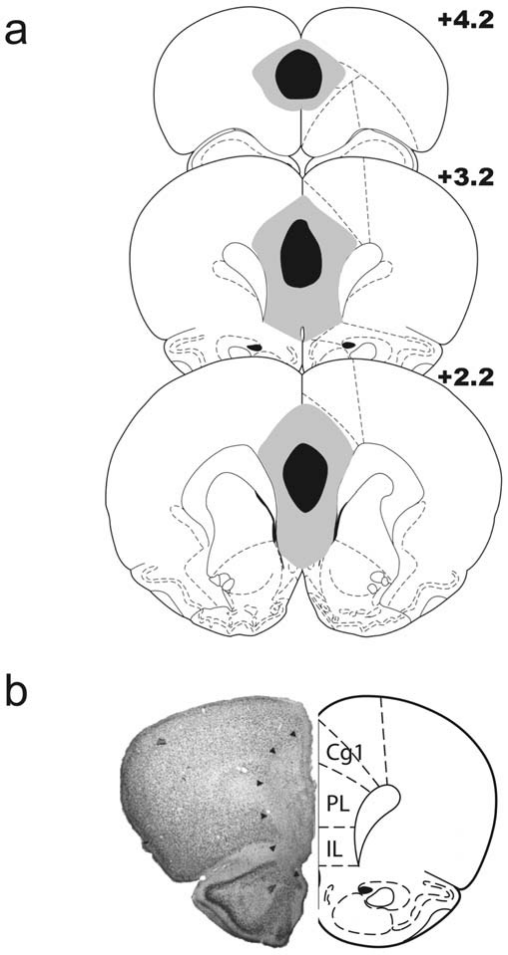
Schematic representation of the extent of medial prefrontal cortex lesions. a) minimal (black area) and maximal (gray area) mPFC lesions affected both the prelimbic and infralimbic parts of the medial prefrontal cortex. b) Photomicrograph of a typical mPFC lesion, illustrating cell loss (outlined by arrowheads). Cg1: Cingulate Cortex 1; PL: Prelimbic Cortex; IL: Infralimbic cortex.

Figure 2 illustrates the extent of the mPFC lesion. For histological analysis, significant cell loss or gliosis in the targeted area and no significant damage to the neighbouring structures were used as criteria for inclusion.

The lesions were found acceptable in 12 rats. As shown, the damaged area primarily concerned the prelimbic and infralimbic cortices. In four rats, the rostral part of the anterior cingulate cortex was also affected. Two rats were discarded because they had only unilateral lesions and therefore their two yoked animals were also discarded from the statistical analysis. The final cell sizes were therefore as follows: SHAM-negative (n = 8), SHAM-null (n = 8); mPFC-negative (n = 6), mPFC- null (n = 6).

#### Instrumental training and baseline responding

Lesioned and control rats acquired the initial instrumental response at identical rates (*Fs*<1 for all effects involving groups) and attained a plateau in instrumental performance after three sessions of training (data not shown).

By the end of training (last VI-60 session), there was no difference in the levels of lever press responding between animals allocated to the various groups. The mean rates of responding were as follows: SHAM-negative: 13.3 responses/min; SHAM- null: 15.7; mPFC-negative: 15.3; mPFC- null: 15.2. An ANOVA with Group (SHAM, mPFC) and protocol (negative, null) revealed no effect of any of the factors (*F*′s<1). Thus, subsequent stages of the experiment were not biased by any difference in baseline responding.

#### Changes in action-outcome contingency

**Figure 3:**
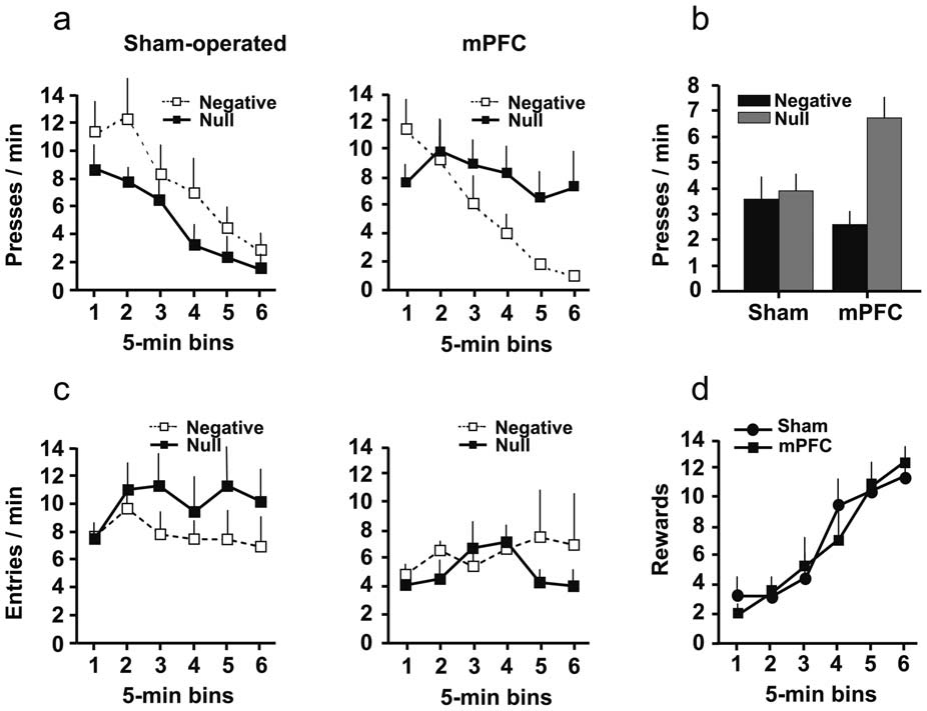
Adaptation to contingency changes. a) Evolution of the rate of lever-pressing during the session of contingency change in blocks of 5 min. (mean + s.e.), according to lesion and condition b) final rate of response at test. Data are expressed as mean rates of responding. c) Evolution of the rate of entries into the empty magazine during the session of contingency change (mean + s.e.). d) Evolution of absolute number of rewards delivered during the session of contingency change in blocks of 5 min., according to lesion. Equal rewards are delivered in both conditions. Negative: negative contingency condition; Null: null contingency condition.

Figure 3a shows the effect of contingency changes on instrumental performance. As shown on the left panel, Sham-operated animals (left panel) gradually learned to withhold lever pressing under the negative contingency condition where lever pressing prevented food delivery, as well as under the null contingency condition where lever pressing had no effect. By contrast, rats with lesions of the mPFC maintained a high level of responding throughout training in the null contingency condition (right panel). However the mPFC-lesioned animals were able to correctly reduce their responding in the negative contingency condition, like sham-operated animals.

Statistical analysis confirmed this description of the data. A mixed analysis of variance with between-subject factors ‘lesion’ (SHAM, mPFC) and ‘condition’ (negative, null) and the within-subject factor ‘acquisition’ (block of 5 min) revealed a significant effect of acquisition (*F*_5,120_ = 21.4, *P*<0.001). More importantly, the analysis showed the existence of a significant three-way interaction (*F*_5,120_ = 2.75, *P* = 0.022), indicating that contingency changes differentially affected lesioned *vs.* intact rats in the negative and null conditions.

Separate analysis of each lesion group indicated that sham-operated animals showed an effect of acquisition (*F*_5,70_ = 14.7, *P*<0.001) but no acquisition×condition interaction (*F*<1). Their performances were therefore comparable in both contingency conditions. In contrast, a similar analysis performed in mPFC-lesioned rats showed a significant effect of acquisition (*F*_5,50_ = 8.34, *P*<0.001) but also an acquisition×condition interaction (*F*_5,50_ = 4.53, *P* = 0.002), with *post-hoc* comparisons revealing that negative and null contingency performances did differ at the end of the session but not at the beginning (*P*<0.05). Indeed, under the null contingency condition, there was no significant decrease in instrumental performance in the mPFC-lesioned group (*P*>0.1), in contrast to sham-operated rats (*P*<0.001).

Delays between lever pressing and food delivery were consistently high in the negative contingency situation, being always 20 s to the first reward delivery, or more if no lever press occurred between rewards. By contrast, in the null contingency situation, these delays were quite variable and sometimes quite short, with a gradually decaying distribution extending to about 15 s. In this situation, mPFC-lesioned rats experienced on average shorter action-reward intervals than control rats (harmonic mean: 0.94 s *vs.* 1.54 s), largely due to their higher response rate.

The results of the test without food delivery are shown in Figure 3b. Again, mPFC-lesioned rats displayed an abnormally high rate of lever pressing in the null contingency condition, and this observation was supported by a significant interaction between ‘condition’ and ‘lesion’ (*F*_1,24_ = 7.05, *P* = 0.014).

Figure 3d shows the gradual increase in food delivery in both groups during the session of adaptation to contingency changes. There was no difference in food delivery between groups (*Fs*<1). Thus, the difference of behaviour between mPFC-lesioned and control groups in the null contingency condition cannot be attributed to a difference in the density of reward.

Figure 3c shows the mean rate of visits to the empty magazine during the contingency-change session. mPFC-lesioned rats displayed a significantly lower magazine activity (*F*_1,24_ = 4.50, *P* = 0.045). There was no evidence of significant changes in magazine entries across the session (*F*_5,120_ = 1.15, *P*>0.1), nor of any effect related to condition or lesion (largest *F* = 1.40, *P*>0.1). In order to further assess the role of response competition in reducing lever-pressing, we evaluated the correlation between rates of magazine entries and rates of lever pressing for each rat over blocks of 2 min (15 measure pairs per rat). The Pearson correlation coefficients between these two measures were highly variable within each group (mPFC-negative: −0.80 to 0.52 ; mPFC-null: −0.38 to 0.53 ; SHAM-negative: −0.41 to 0.87 ; SHAM-null: −0.65 to 0.90). Only three negative correlations and five positive correlations were significant (two-tailed threshold: −0.514). Thus, the decrease of lever-pressing performance, when present, was not necessarily associated with an increase of other behaviours such as waiting at the food magazine.

The occurrence of non-contingent rewards elicited in all groups of rats a visit to the magazine and consumption of the food pellet, after which lever pressing resumed. We found no evidence for a differential pattern of response in the lesioned group, as would be expected if food delivery contributed to energize instrumental responding specifically in this group.

## Experiment 2

The negative and null contingency conditions were characterized by different distributions of delays between lever pressing and reward delivery. Thus, the detection of changes in contingency might depend upon the degree of temporal contiguity between response and outcome. The aim of Experiment 2 was therefore to determine whether mPFC-lesioned rats were impaired in detecting changes in contiguity between an action and its outcome.

### Materials and Methods

#### Subjects

Thirty two male, Long Evans rats obtained from Centre d'Elevage Janvier (France) were used. Housing and surgical procedures were identical to those used in Experiment 1 except that surgery was performed under isoflurane anaesthesia and NMDA was injected by means of a pressure ejection system (Picospritzer, General Valve Co., Brookshire, TX). The rats were allocated to the various groups in a fully counterbalanced manner. Half the rats (8 mPFC, 8 SHAM) were naïve and were included in the experiment immediately following recovery, at a weight of 320–390 g. The other half (weighting 380–515 g) had undergone surgery two months earlier and had then been submitted to a Pavlovian to instrumental transfer experiment which included appetitive Pavlovian and instrumental training. No statistical difference between these two subgroups was found in any of the target analyses.

#### Behavioural procedures

For initial lever press training, the naïve rats underwent two days of magazine training, then were trained for 2 sessions under a continuous reinforcement (FR-1) schedule, in which one food pellet could be obtained with each lever press. A session ended as soon as 60 rewards were earned or after 45 minutes had expired. All rats were then switched to four sessions of variable-interval 30 s schedule (VI-30).

After the initial training, the action-outcome contiguity was altered. Each group of rats was distributed into a delay (n = 10) and a no-delay (n = 6) condition. Rats in the no-delay condition received a single session of VI-30 as before, with the exception that session ended after 69 rewards were earned or after 120 minutes had expired. Rats in the delay condition were submitted to delays between lever press and pellet delivery that increased gradually from 0 to 8 s. For these rats, a VI-30 schedule was also in effect but a delay was inserted prior to each reward delivery. Lever pressing during this delay reset the delay, thus postponing the reward and ensuring strict application of the programmed delays. A resetting event was thus characterized by the occurrence of two lever-presses closer together than the programmed delay, followed by a delayed reward. The first four rewards were delivered without a delay, and delay was incremented by 0.5 s after each sequence of four rewards, i.e. 0.5 s delay for rewards 5–8, 1 s delay for rewards 9–12 etc. Thus, no reward could be obtained in the absence of lever pressing, but the gradually increasing delay in the delayed group disrupted action-outcome contiguity [15] and resulted in response and outcome appearing unrelated.

On the next day, the rats were exposed to a new test session with delays varying in the opposite direction, i.e. starting with the maximal delay and ending with no delay.

#### Data analysis

Rates of lever pressing and magazine entries were calculated over blocks corresponding to each delay or over consecutive blocks of four rewards in the no-delay group. Statistical analyses were performed using ANOVA with lesion (Sham, mPFC) and condition (delay *vs.* no-delay) as between subject factors and blocks as repeated measures. The alpha value for rejection of the null hypothesis was 0.05 throughout.

### Results

#### Histology

The extent of the mPFC lesion was similar to that in Experiment 1, and therefore all histological data were collapsed as illustrated in figure 2. The lesions were found acceptable in all the rats in this experiment with damage to the prelimbic and infralimbic regions, as well as partial damage to the rostral part of the anterior cingulate cortex in all of the rats. The final cell sizes were therefore as follows: SHAM-delay (n = 10), SHAM-no-delay (n = 6); mPFC-delay (n = 10), mPFC-no-delay (n = 6).

#### Instrumental training and baseline responding

Lesioned and control animals learned the instrumental response at identical rates over the 6 sessions of training (data not shown). By the last session of training, there was no significant difference in the levels of lever press responding between animals allocated to the various groups (*F*′s<1). The mean rates of responding were as follows: SHAM-delay: 14.4 responses/min; SHAM-no-delay: 15.9; mPFC- delay: 16.1; m-PFC-no- delay: 12.6.

#### Changes in action-outcome contiguity

**Figure 4:**
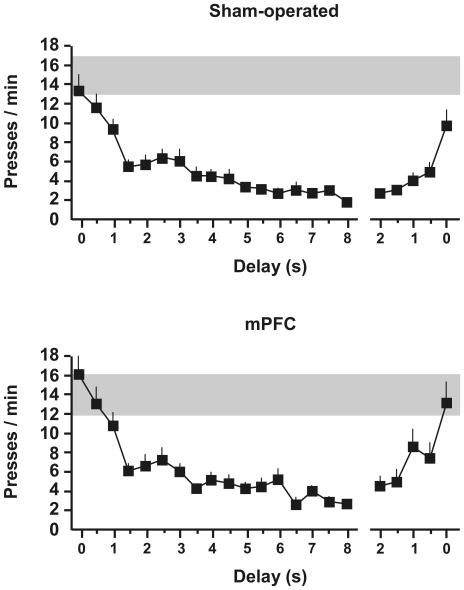
Adaptation to contiguity changes in sham and mPFC-lesioned rats. Upper panel: Sham, control rats. Lower panel: PFC: rats with lesions of the medial prefrontal cortex. Data points represent average lever-pressing rate across blocks of fixed delay (mean + s.e.). Delay between lever press and reward was increased by 0.5 s after each block of four rewards. Last data points show the recovery of responding with short delays during test on the next day. Grey area shows range of values observed in group no-delay across the whole session (computed over blocks of four rewards).

Figure 4 shows the evolution of instrumental performance in groups submitted to increasing delays of reward or to no delay. Both mPFC-lesioned and Sham rats maintained stable and comparable levels of responding throughout the session when rewards were not delayed. By contrast, lever pressing rates sharply decreased with increasing delays in both Sham and mPFC groups. Performance already dropped to less than 50% when rewards were delayed by 2 s. Lever pressing rates then continued to decrease gradually with longer delays down to about 20% of those in the no-delay groups. The lesioned and Sham groups displayed very similar adaptation curves. Furthermore, these effects were reversible (Figure 4, last data points in each panel). Long delays to reward maintained very low response rates, but all but one rat from each group showed a rapid reappearance of responding when delays were reduced to 1 s or less.

A repeated-measures ANOVA on the results from increasing delays showed a clear effect of condition (*F*_1,28_ = 67.2, *P*<0.001) that interacted with delay (*F*_15,420_ = 14.7, *P*<0.001). Separate analyses of conditions revealed a clear effect of delay in the delay groups (*F*_15,270_ = 47.2, *P*<0.001) and not in the no-delay groups (*F*_15,150_<1), but no difference between Sham and mPFC groups nor any interaction with delay in either condition (all *Fs*<1). A more detailed analysis conducted on short delays (0.5 to 4 s) led to the same conclusions. Thus, mPFC-lesioned rats appear unimpaired in adapting their instrumental response to changes in the contiguity between lever-pressing and reward. Recovery on the next day was equally observed in Sham and mPFC rats (delay: *F*_4,72_ = 26.9, *P*<0.001 ; interaction *F*<1), although control rats globally tended to press less (*F*_1,18_ = 3.03, *P* = 0.10).

A similar analysis was conducted on magazine entry behaviour during this session (Data not shown). Rates of magazine entries appeared overall stable and comparable between groups (SHAM-delay: 9.8 responses/min; SHAM-no-delay: 10.4; mPFC-delay: 9.7; m-PFC-no-delay: 11.6). The only significant effect was an effect of delay (*F*_15,420_ = 2.39, *P* = 0.0025) limited to the delay groups (*F*_15,270_ = 3.48, *P*<0.001), irrespective of lesion (all other *Fs*<1.23). This effect was due to a significant but transient increase in magazine entries when the delay became equal to 2 s. Therefore, the marked decrease in lever press behaviours induced by delays was not accompanied by any lasting change in magazine activity.

Finally, we also evaluated the number of rewards which were postponed by the resetting delays in the delay groups. The resetting delay procedure ensured that the actual response-reward delays were just those programmed, but at the cost of a possible increase in the overall interval between rewards. For delays of up to 2 s, mPFC and SHAM rats experienced on average 1 and 2 resetting events per rat, respectively, over 20 rewards and approximately 155 lever-presses. Actually, two mPFC rats and four SHAM rats did not experience any resetting event over this period. No clear relationship could be observed between the occurrence of these events and the decrement in lever-press activity.

## Discussion

The present study demonstrates that rats with lesions of the mPFC are capable of some adaptation to changes in instrumental contingency or contiguity. In Experiment 1, mPFC-lesioned rats remained able to learn the shift to a negative contingency as well as normal rats. However, in the null contingency condition, they failed to decrease their rates of lever pressing. In Experiment 2, mPFC-lesioned rats demonstrated a normal sensitivity to changes in contiguity, with an ability to detect reinforcement delays of about 2 s and a rapid reduction in their rates of responding. These findings have important implications for mPFC functions as discussed below.

### Specificity of the Effects

A number of features in Experiment 1 preclude trivial explanations of these findings. All groups obtained similar amounts of food during the session. In addition, previous research has established that lesions of the mPFC do not affect consummatory responding [8], [16], [17].

Rates of acquisition of the instrumental task were not affected by these lesions [7], [8]. Similarly, although extinction is likely to be an important factor in negative contingency training [18], there was no evidence for any difference in extinction rate in the mPFC-lesioned animals, both in our experiments under the negative contingency condition and in previous reports (e.g. [5]). In addition, the lesioned rats did not appear to be more prone to lever pressing or to over-sample the environment [19] since baseline rates of response were unaffected by the lesion.

The negative contingency condition (omission) is also known as DRO (differential reinforcement of other behaviours [20]. Thus, other (unrecorded) behaviours might have been reinforced during delays and competed with lever pressing. No such learning would be possible in the null contingency condition. For instance, changes in reward-elicited approach behaviour might have occurred. However, changes in magazine entries were not negatively correlated to lever pressing and were not significantly different in mPFC-lesioned rats under the two conditions (See also [21]). Moreover, the difference between groups persisted in the subsequent test without food, indicating that learning processes rather than response competition effects were responsible for the observed differences.

mPFC-lesioned rats did differ from control rats in their overall rates of magazine entries during this experiment. However, this was not a result of contingency changes. Indeed, further analyses revealed a gradual appearance of this effect during the instrumental training phase. Moreover, this difference in magazine entries essentially concerned magazine visits occurring just after lever presses [7], suggesting that normal rats are more likely than mPFC-lesioned rats to chunk these two actions into a single behavioural unit [22].

The similar adaptation of the control groups in the two contingency conditions may be considered surprising, as it is sometimes argued that noncontingent reinforcement should produce little response rate reduction in normal subjects [23], [24]. However, several factors appear to influence the effectiveness of a noncontingent procedure: for instance, a difference between negative and null contingency training often becomes apparent only after a number of test sessions [25], [13]. Moreover, noncontingent schedules with inter-reinforcer intervals longer or shorter than baseline appear to be particularly effective in reducing responding [26]. In our study, the large response decrement may have in part resulted from the low initial reward rate in both conditions.

Finally, lesioned rats experienced shorter intervals between response and outcome than control animals in the null-contingency condition. This may be a result as well as a cause of persistent lever-pressing. In the present and similar experiments [21], [25], non contingent conditions may lead to some pseudo-contingent rewards occurring by chance. This may contribute to maintain the contingency between response and outcome [27] and could specifically support responding in lesioned animals.

### The mPFC and Habitual Responding

Using an identical protocol (except for three more sessions of FI20 training), Yin *et al.* [21] found no difference between the omission and yoked treatment in their control groups. They interpreted this as evidence for Stimulus-Response (habitual) responding - although responding in both groups appeared to decrease by half across the session. In the present experiments, control rats showed a marked decrement of responding in the null contingency (yoked) condition, as well as persistence of this effect in extinction, suggesting that they were sensitive to the consequences of their action.

Yin *et al.* [21] also observed that inactivating the dorso-lateral striatum (DLS) largely suppressed responding during omission and yoked training and revealed a difference between groups during the subsequent test in extinction. In our experiments, mPFC lesions did not affect the overall rate of lever-pressing, but induced a deficit of adaptation in the yoked condition both during training and during the subsequent test in extinction. In this respect, mPFC and DLS inactivation appear to have similar effects. This is unusual since DLS inactivation is considered to favour goal-directed over habitual responding [21]. By contrast, mPFC lesions are thought to prevent the acquisition of goal-directed behaviour [5], [6]. Although our lesions encompassed both the prelimbic and infralimbic regions, the latter being involved in controlling habitual behaviour [28], previous studies suggest that prelimbic lesions, but not damage to the infralimbic region, may be sufficient to produce the present pattern of results [8], [9].

Therefore, it may be suggested that an absence of difference between omission and yoked groups is not sufficient to characterize habitual responding, at least when responding decreases in both conditions. However, mPFC-lesioned rats appear to express habitual responding [29] when they fail to adapt to a null contingency under yoked training.

### The mPFC and Contiguity

The deficit of mPFC-lesioned rats in contingency detection might be related to an altered perception of the temporal relationship between response and outcome, in agreement with the involvement of prefrontal areas in cross-temporal associations [30]. However, Experiment 2 shows that lesioned rats perceive action-outcome delays in a normal manner and indicates that contiguity *per se* may not be a critical determinant of response rates in lesioned rats. When faced with a gradual disruption in the contiguity between lever press and reward, mPFC-lesioned animals quickly reacted by reducing their lever presses in the same way as control rats. No effect of mPFC lesions could be observed, and adaptation of lever pressing rates occurred with delays as short as 1.5–2 s.

Experiment 2 differs from a test of omission in several aspects: firstly, most of the adaptation occurred with much shorter delays than those used in omission experiments. For delays up to 2 s, the small number of resetting events makes this condition virtually undistinguishable from a non-resetting delay. Secondly, unlike in an omission schedule, the overall relationship between response rate and reward rate was not negative. Indeed, low rates of responding should delay the reward, and high rates of responding should not, as long as the proportion of resetting events remains small.

Although the effects of delay are confounded with those of increasing experience with delayed rewards during the session, these effects were reversed by removing the action-outcome delay. This confirms the flexibility of behaviour in mPFC-lesioned rats and indicates that delays, rather than learning, were the major determinant of response decrement. Such an effect of delay was reported long ago in normal animals [31] and it is thought to reflect a form of causality judgement [15]. That is, the rats may press the lever less in the presence of delays because they are less sure that this action is responsible for reward occurrence. Alternatively, rewards delayed by a few seconds could be considered less valuable [32], an effect that has been observed to increase with dopamine depletion of prefrontal regions such as the orbitofrontal cortex [33]. However, no such effect was observed here with medial prefrontal lesions. Finally, working memory processes [34] probably did not contribute much to these effects since the delays considered in the contiguity experiment were quite short.

### The mPFC and Contingency

Experiment 1 agrees with previous research demonstrating that the mPFC is necessary to adapt to contingency degradation [9], [10], [29]. However, it also demonstrates that animals with lesion of the mPFC remain capable of adaptation.

To understand the deficit in mPFC-lesioned animals, let us consider the differences between the null and negative contingency conditions. In null contingency condition, the rate of reward delivery is unrelated to the rate of instrumental response, the timing between response and reward is random and short response-outcome intervals may occur by chance. By contrast, in the negative contingency condition, the rate of reward delivery is negatively related to response rate and the delay between response and reward is consistently long.

The adaptation of mPFC-lesioned rats to a negative contingency is not simply reducible to the optimization of behaviour with respect to reward rate, since this cannot account for the performance of these rats in the delayed reward task of Experiment 2. Indeed, a lower response rate in this task reduces rather than increases the amount of reward obtained.

It may be noticed that in both experiments, imposing a delay between response and reward may amount to a punishment contingency, possibly resulting in frustration [35]. Therefore, we cannot fully exclude that such a contingency might prevent the appearance of a deficit in mPFC-lesioned rats, to be revealed in the absence of such a negative contingency.

Another possibility would be that normal rats, but not mPFC-lesioned rats, are sensitive to the overall correlation between action and reward, and are therefore able to suppress responding in the null contingency condition. On a coarse-grained (molar) scale, they might observe periods of high response rates associated with low reward rates and/or the reverse, but this would require the integration of events over a very long time scale because of the low reward rates. Moreover, the variable interval schedule by itself tends to break the correlation between response and outcome [36] and should therefore tend to produce lower response rates in normal subjects.

This leads us to focus our interpretation on fine-grained (molecular) determinants of learning, i.e. the precise temporal relationship between responses and rewards. Experiment 2 demonstrates that all rats clearly differentiate short (i.e. <2 s) from long (>2 s) response-reward delays. Short delays are perceived as contingent rewards and maintain instrumental performance, presumably by preventing extinction of the action-reward association. Long delays will be perceived as non-contingent rewards. They reduce performance in all rats, as shown in Experiments 1 and 2, provided short delays are absent.

There is no indication that mPFC-lesioned rats might be more sensitive to chance pairings that occur during null contingency training. In a related article [7], we investigated the difference between normal and mPFC-lesioned rats using temporal-difference learning. We found that both groups could be described with similar parameters for perception and learning. This is consistent with the view that model-free reinforcement learning does not require prefrontal involvement and may be implemented in the dorsal striatum [37]. These simulations based on actual behavioural data indicate that mPFC respond to fortuitous short response-outcome intervals in a similar way as control rats.

However, it appears that mPFC-lesioned rats show a deficit when both long and short delays are combined. A likely interpretation of our results is therefore that the mPFC is involved in evaluating the balance between contingent and non-contingent reinforcement, which is at the core of contingency computation. In normal rats, the decision to press the lever might depend upon an outcome-specific comparison process [9], [10], [11], [29], whereas lesioned rats may only rely upon a general value attributed to other actions or the context. Indeed, cortical model-based systems that explicitly encode event consequences in the form of state transition probabilities [38], [39] might be more efficient than model-free reinforcement learning under variations in action-outcome contingency.

The neural basis of these operations in the mPFC is still poorly understood. Dopaminergic mechanisms have been reported to play an important role in flexible, goal-directed instrumental behaviour [40], [41]. Furthermore, we have recently shown that lesions of dopaminergic terminals or D1/D2 receptors blockade within the prelimbic area of the mPFC prevent the adaptation to contingency degradation [11] (but see [42]). This is consistent with the notion that unpredicted, non-contingent rewards elicit a dopaminergic prediction error signal. As dopaminergic signals in the mPFC appear to have more tonic effects than in the striatum [43], they may be appropriate to integrate the amount of non-contingent reinforcement and to prompt a change in behaviour. Moreover, variability of delays between action and reward induces ramping of dopamine activity [44] and the mPFC may be especially important in detecting these signals.

### Conclusions

Studies in monkeys and humans have revealed activity in prefrontal regions that track changes in contingency [3], [45], suggesting that similar processes in the mPFC of rodents and primates underlie the evaluation of actions consequences and the subsequent behavioural adaptation [3]. We show here that mPFC-lesioned rats are capable of flexible behaviour in omission as well as in response to short delays between action and reward. Since these findings impose constraints on the role of the mPFC in instrumental behaviour, they might lead to a reappraisal of the classical view of habitual behaviour induced by prefrontal lesions [9] and the role of this region in behavioural flexibility [46].

The mPFC appears required when actions become unrelated to their outcome, i.e. when they lose their causal status. This function could be based on the precise temporal relationship between responses and rewards rather than on their molar statistical properties. It has been proposed that uncertainty computed in the mPFC regulates the balance between goal-directed and habitual actions [47]. The present data suggest that variability in action-outcome delays may induce uncertainty in intact, but not mPFC-lesioned rats, and thereby regulate the context for decision making [19], [48].
